# *Gastrocoptaarmigerella* (Reinhardt, 1877) and *Gastrocoptatheeli* (Westerlund, 1877) in western Tien Shan, Kyrgyzstan, and their further occurrence in Asia (Mollusca, Gastropoda, Pupilloidea)

**DOI:** 10.3897/zookeys.807.29113

**Published:** 2018-12-17

**Authors:** Stefan Meng, Ulrich Bößneck

**Affiliations:** 1 Institute of Geography and Geology, University of Greifswald, Friedrich-Ludwig-Jahn-Straße 17a, D-17489 Greifswald, Germany University of Greifswald Greifswald Germany; 2 Natural History Museum Erfurt, Große Arche 14, D-99084 Erfurt, Germany Natural History Museum Erfurt Erfurt Germany

**Keywords:** Asia, *
Gastrocopta armigerella
*, *
Gastrocopta theeli
*, Pupilloidea, Tien Shan

## Abstract

*Gastrocoptaarmigerella* (Reinhardt, 1877) has been described from Japan and is widespread in the Far East and China. Surprisingly, a few occurrences in central and western Asia have also become known. Forcart (1935) found *G.armigerella* in northern Iran. The authors found evidence of *G.armigerella* in western Tien Shan, Kyrgyzstan. The form from northern Tajikistan described by Schileyko (1984) as *G.huttoniana* agrees morphologically with *G.armigerella* as well. *Gastrocoptahuttoniana* is known from western India and the Himalayan region. The evidence of *G.armigerella* from central and western Asia has come thus far from drift material at the high water line in river floodplains which suggests that these are sub-fossil or fossil shells (Holocene or Pleistocene) which have been relocated. No living example of *Gastrocopta* has been found there as yet. Possibly the species is now extinct in this region. *Gastrocoptatheeli* (Westerlund, 1877) is the most widespread *Gastrocopta* in Eurasia. Its area ranges from the Caucasus to the Far East. The findings reported here are the first for this species in western Tien Shan.

## Introduction

*Gastrocoptaarmigerella* (Reinhardt, 1877) type locality Misaki (Japan), has been reported from Japan (Möllendorff 1901; Nekola et al. 2012; Nishi et al. 2017; WMSDB) and China: Gansu, Batang, eastern Tibet, Hunan, Xifeng, Yangtze region, Anhui, Zikarvei, Dshiangsu, Shandong, Shanghai (Möllendorff 1901; Pilsbry 1916–1918; Yen 1939; Chen and Gao 1987; Wu et al. 2007; Zhouxing and Deniu 2008; Pokryszko and Stworzewicz 2004; WMSDB). *Gastrocoptaarmigerella* has also frequently been found in a fossil state (Pleistocene) (e.g., Wu et al. 2007; Wu and Wu 2011) on the Chinese loess plateau (Xifeng and Luochuan). In addition, *G.armigerella* was reported from Korea (Möllendorff 1899; Pilsbry 1916–1918; National Institute of Biological Resources 2012). However, Pokryszko and Stworzewicz (2004), for example, doubt its occurrence in North Korea and suspect a confusion with *Gastrocoptahirasei* Pilsbry 1916, a synonym, which Pilsbry (1916–1918) originally described from China (see below).

Forcart (1935) surprisingly found *G.armigerella* in northern Iran near Meshhediser (Mazenderan Province) on the river floodplain in drift material from the Babul River not far from the coast. Because the shells were slightly smaller, he described these as a new sub-species *G.armigerellamasenderanensis*.

*Gastrocoptahuttoniana* is know from western India (northeast of the western Ghats, west and central Maharashtra) and the Himalayan region (Pakistan, northern India, Nepal) (Pilsbry 1916–1918: 137, pl. 21, fig. 16–17; Kuznetsov and Schileyko 1997; Mitra et al. 2005; Pokryszko et al. 2009: 433, 434, fig. 26, 27; Ramakrishna et al. 2010; Raheem et al. 2014; Budha et al. 2015; Bößneck and Meng 2018).

Schileyko (1984, with reference to Izzatullaev 1970) and subsequently Kantor and Sysoev (2005), Sysoev and Schileyko (2009), and Egorov (2008) refer to *Gastrocoptahuttoniana* (Benson, 1849) from northern Tajikistan, south of the Hissar Mountains near Dushanbe, in drift material from the Kafirnigan River. On this matter, the authors present new data from western Tien Shan.

*Gastrocoptatheeli* (Westerlund, 1877), type locality Mikoulino/Yeniseysk (Russia), is the most widespread *Gastrocopta* in Eurasia. Relict populations have been found in the Caucasus region: on the floodplains of the Kura and Rioni Rivers (Georgia), in Dagestan and the north Caucasus (Russia); as well as elsewhere in Russia and adjacent countries: in Chelyabinsk and Yeniseysk, central and southern Altay (Russia and Kazakhstan), Kyrghyz-Ala-Too in north west Tien Shan (Kazakhstan/Kyrgyzstan), in South Primorskij Kraj (Pilsbry 1916; Schileyko 1984; Uvalieva 1990; Egorov 2008; Sysoev and Schileyko 2009; Schileyko and Rymzhanov 2013), in Tuva (Zasypkina 2012); in the Middle Amur River basin (Prozorova et al. 2014), in Holocene deposits from the upper Lena River in the Lake Baikal region (White et al. 2008); in Japan (e.g. Naohiro 2015; Nishi et al. 2017) and the Korean Peninsula (Prozorova et al. 2014).

**Figure 1.**
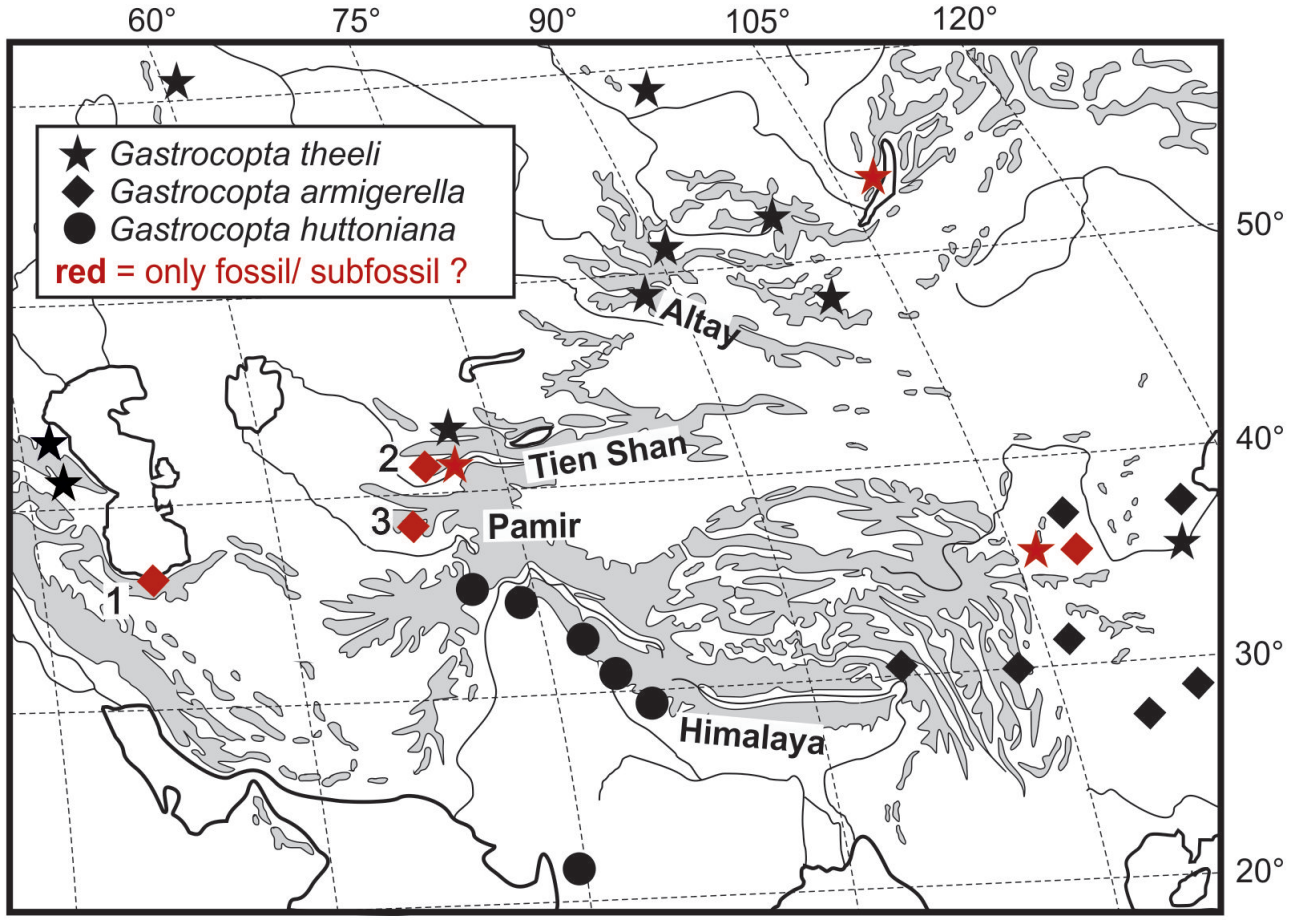
Map with locations of *Gastrocoptatheeli, armigerella* and *huttoniana* in central Asia and adjacent territories; *G.armigerella*: **1** northern Iran, Babul River near Meshhediser, Mazenderan Province (Forcart 1935) **2** Kyrgyzstan, western Tien Shan, Kara Suu River near Dshany Dshol **3** northern Tajikistan, southern Hissar Mountains, Kafirnigan River near Dushanbe (Schileyko 1984).

As *G.coreana* Pilsbry, 1916, *G.theeli* has been reported from China: Tsinan Fu, Shangdong (Yen 1939), fossil (Pleistocene) from the Chinese loess plateau (e.g. Wu et al. 2007; Wu and Wu 2011); from Japan (e.g. Pilsbry 1916–1918) to North and South Korea (e.g. Pilsbry 1916–1918; Pokryszko and Stworzewicz 2004; National Institute of Biological Resources 2012). *Gastrocoptacoreana* Pilsbry 1916, type locality Kojeto (South Korea), is today often considered to be a synonym of *G.theeli* (e.g. Schileyko 1984; Prozorova et al. 2014; WMSDB). New data on the occurrence of *G.theeli* from the Altay, Tien Shan, Mongolia, and the Far East are submitted by the authors (Fig. 1).

## Materials and methods

From the Kara Suu River floodplain in the western Tien Shan near Dshany Dshol (Kyrgyzstan), 860 m a.s.l., 41°35'05.8"N, 072°08'03.3"E, 21.07.1998, leg. S. Meng, the authors have a sample of river drift material from the high water line containing approximately 2000 gastropod shells in total. The genus *Gastrocopta* is predominant with ~ 430 shells. The *Gastrocopta* shells are probably all of fossil or sub-fossil origin. *Gastrocoptaarmigerella*, *G.theeli* and *G.huttoniana* are also evaluated in the bio-geographical context. Additional records of *G.theeli* in the Siberian Altay and the Far East collected by S. Meng are also included.

## Results

*Gastrocoptaarmigerella* was found in the drift material from the Kara Suu River floodplain near Dshany Dshol, 860 m a.s.l. (western Tien Shan, Kyrgyzstan). The community as a whole contained mainly elements of the modern thermophilous communities of lowlands and large mountain valleys. The predominant forms were, e.g., *Vertigopygmaea* (Draparnaud, 1801), *V.antivertigo* (Draparnaud, 1801), *Truncatellinacallicratis* (Scacchi, 1833) or *Valloniapulchella* (Müller, 1774). The shells of *G.armigerella* (Figs 6–8) comprise ~ 20 %, more than 400 shells, of the sample. No remnants of organic material, such as tissue or periostracum were found in any of the *G.armigerella* specimens. Because of their preservation it is assumed that the shells do not belong to the modern faunal communities and that the material is of Holocene or Late Pleistocene age. Three shells of *G.theeli*, probably fossils as well, were also found (Fig. 5). This is also a new record for the western Tien Shan. Two fossil or sub-fossil shells of *Valloniazaru* Almuhambetova, 1979, a species today restricted as a relict to the northern Tien Shan, were found as well.

**Figures 2–5.**
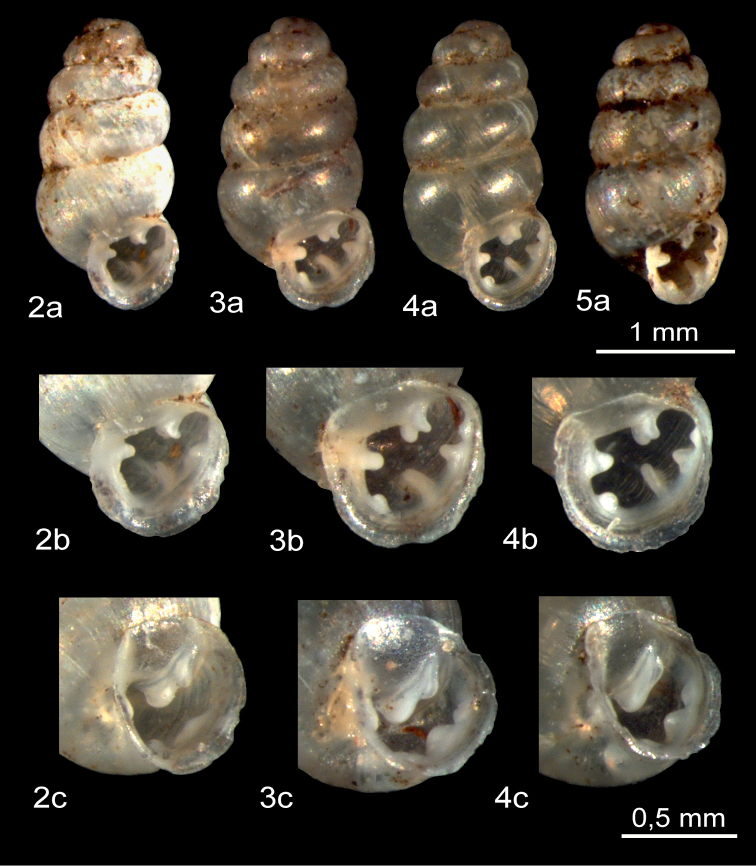
*Gastrocoptahuttoniana***2 a–c** Himalaya, Simikot (District Humla/ Province Karnali, western Nepal), leg. U. Bößneck, 2001; *Gastrocoptatheeli***3–5**: **3 a–c** western central Altay, southeastern Ongudai, (Russia) **4 a–c** South Primorskij Kraj, south of Slawjanka, coast of Sea of Japan (Russia) **5 a** western Tien Shan Mountains, Kara Suu River near Dshany Dshol (Kyrgyzstan).

**Figures 6–8.**
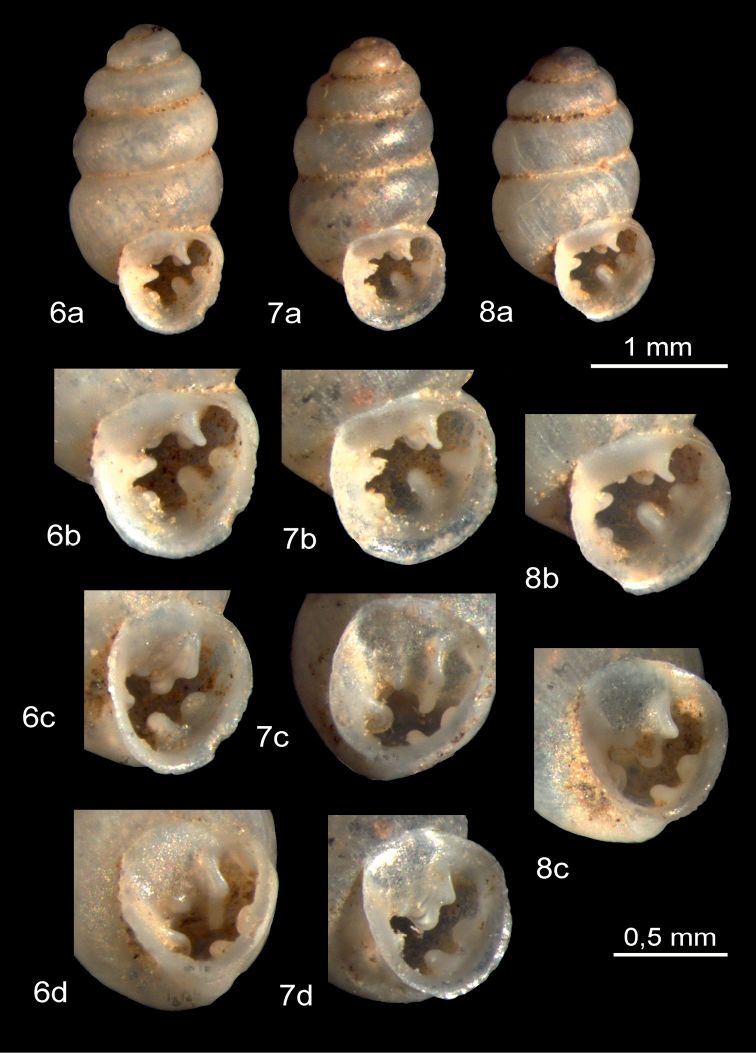
*Gastrocoptaarmigerella*: western Tien Shan, Kara Suu River floodplain (drift material) near Dshany Dshol (Kyrgyzstan), fossil or sub-fossil (Holocene or Pleistocene).

The shell morphology of *G.armigerella* from western Tien Shan (Figs 6–8) corresponds to the descriptions and figures of Reinhardt (1877: pl. 11, fig. 7), Pilsbry (1916–1918: pl. 21, fig. 1 as *G.a.hachijoensis*, pl. 21, fig. 5, 6, 7, 10 as *G.hirasei*), Forcart (1935: 421, fig. 2 as *G.* a. m*asenderanensis*), Schileyko (1984: 197, fig. 114II as *G.huttoniana*), Chen and Gao (1987: 42, fig. 50), Pokryszko and Stworzewicz (2004: 135, fig. 3 as *G.hirasei*), Sysoev and Schileyko (2009: fig. 19 F as *G.huttoniana*), Nekola et al. (2012: 49, fig. 1) and Nishi et al. (2017: 27, fig. 2).

**Figure 9.**
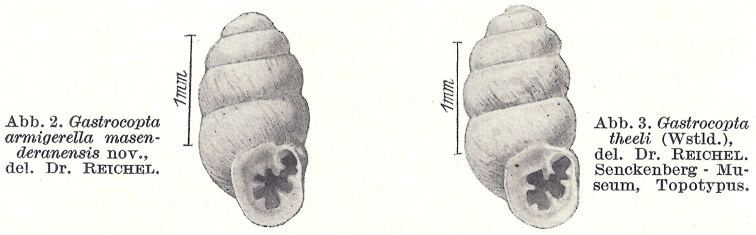
Original figure by Forcart (1935, p. 421), *Gastrocoptaarmigerellamasendarensis* from Meshhediser (northern Iran) in comparison with *Gastrocoptatheeli*, topotype (shell from type locality Yeniseysk), Senckenberg Museum.

*Gastrocoptaarmigerella* has five markedly convex whorls. Compared with *G.huttoniana* and *G.theeli*, its apertural dentition is very strongly developed (Figs 2–9). *Gastrocoptaarmigerella* usually has 7–8 apertural teeth. The angular-parietal lamella has two calluses which are not completely fused. The front tip is inclined towards the palatal wall. An infraparietal (subparietal) callus is present. The columellar tooth forms a strong lamella. The columellar/basal callus is also well developed. There are two palatal lamellae, the lower one of which is more pronounced. In the suprapalatal position there is often a slight thickening of the apertural lip (Reinhardt 1877; Pilsbry 1916–1918; Forcart 1935).

In contrast, *G.theeli* has no infraparietal tooth, the angular-parietal lamella is almost completely fused and three lamellae are found on the palatal wall (Figs 3–5). In general, *G.theeli* and *G.armigerella* do not vary much and are relatively stable in their respective shell morphologies.

The *Gastrocopta* material from northern Tajikistan (Schileyko 1984; Sysoev and Schileyko 2009) corresponds morphologically to *G.armigerella*, including *G.armigerella* specimens from western Tien Shan and northern Iran (Forcart 1935) (Fig. 9). The shell sizes (2–2.3 × 1.1–1.2 mm) given by Schileyko (1984) also correspond to the material from western Tien Shan (2.1–2.5 × 1.1–1.2 mm). Since *G.armigerella* was only found in river drift material and never alive in northern Iran and northern Tajikistan, it must be assumed that this material is also fossil or sub-fossil.

In view of the breadth of its morphological variation, the definition of *G.huttoniana* is more problematic (Bößneck and Meng 2018). In the lectotype and paralectotypes from Simla in northern India (Raheem et al. 2014: fig. 37A, B), the columellar/basal callus is much reduced and merely indicated and there is a complete lack of an infraparietal tooth (Fig. 2). However, Pokryszko et al. (2009: 434, fig. 26–28) assumes for the *G.huttoniana* material from Pakistan that a small infraparietal callus is often present. Forms with an infraparietal callus are also known from Nepal but the systematic status of the material is uncertain (Bößneck and Meng 2018).

*Gastrocoptatheeli* from western Tien Shan (Fig. 5) corresponds in its shell morphology to the descriptions and figures of Westerlund (1877: 113, fig. 4), Pilsbry (1916–1918: pl. 21, Figs 2–4, as *G.coreana*), Forcart (1935: 421, fig. 3, topotype), Schileyko (1984: 197, fig. 114I), Uvalieva (1990: 113, fig. 8), Sysoev and Schileyko (2009: 333, fig. 19G, paralectotype), Schileyko and Rymzhanov (2013: 399, pl. 5B, syntype of *Pupa denudata* Mousson 1887), White et al. (2008: 971, fig. 6i), and Pokryszko and Stworzewicz (2004: 135, fig. 3, as *G.coreana*).

In addition to the evidence of *G.theeli* in western Tien Shan, the authors can list the following further localities which confirm the main areas of occurrence of the species in the Altay and Far East (Fig. 1).

**Tien Shan, Kyrgyzstan**: western Tien Shan, Kara Suu River floodplain (drift material) near Dshany Dshol, 860 m a.s.l., 41°35'05.8"N, 072°08'03.3"E, 21.07.1998, leg. S. Meng, subfossil (?).

**Altay, Russia**: western central Altay, near Černyj Anuj, Ust’ Kanskiy Rayon, grassland, subsoil carbonate, 697 m a.s.l., 51°23'17.10"N, 084°41'16.98"E, 04.08.2006, leg. S. Meng; central Altay, Onguday, eastern edge of town, dry grassy slope, under stones, ca. 850 m a.s.l., 50°45'N, 086°10'E, 17.07.1997, leg. S. Meng; Ursul River valley southeast Onguday, feather grass steppe on diabase rock, 747 m a.s.l., 50°43'38.88"N, 086°18'38.52"E, 05.08.2006, leg. S. Meng; feather grass steppe on diabase rock, 750 m a.s.l., 50°43'34.86"N, 086°18'32.52"E, 05.08.2006, leg. S. Meng; herb-rich meadow, 754 m a.s.l., 50°43'33.30"N, 086°18'33.78"E, 05.08.2006, leg. S. Meng; steppe slope, 754 m a.s.l., 50°43'27.00"N, 086°18'24.72"E, 05.08.2006, leg. S. Meng.

**Mongolia**: western Mongolia, Khar Us Lake National Park, rocks, 1200 m a.s.l., 48°20'N, 092°17'E, 30.07.1999, leg. M. Unruh.

**Primorskij Kraj, Russia**: South Primorskij Kraj, south of Slawjanka, coast of Japan Sea, south of Kap Krasnyj Utes, rock cliff, grasses and *Artemisia*, 10 m a.s.l., 42°46'43.2"N, 131°16'01.3"E, 21.07.2012, leg. S. Meng; meadow slope, 15 m a.s.l., 42°46'43.4"N, 131°15'54.4"E, 22.07.2012, leg. S. Meng; South Primorskij Kraj, Island Rikorba, Peter the Great Bay, south of Vladivostok, southwestern area of the island, rock cliff, grasses and *Artemisia*, 10–20 m a.s.l., 42°51'51.7"N, 131°38'23.9"E, 28.07.2012, leg. S. Meng; southwestern area of the island, shrubs meadows on rocks, *Artemisia*, e.g., 30 m a.s.l., 42°52'04.3"N, 131°38'38.3"E, 29.07.2012, leg. S. Meng; southwestern area of the island, shrubs meadows on rocks, *Artemisia*, e.g., 30 m a.s.l., 42°51'47.7"N, 131°38'25.4"E, 29.07.2012, leg. S. Meng; southwestern area of the island, rock cliff, in grasses, 20 m a.s.l., 42°51'48.5"N, 131°38'22.4"E, 31.07.2012, leg. S. Meng; eastern area of the island, dry grassland, rock cliff, 90 m a.s.l., 42°51'54.6"N, 131°39'32.3"E, 31.07.2012, leg. S. Meng.

## Discussion

The evidence of *G.armigerella* and *G.theeli* from the western Tien Shan is of great biogeographic significance. It has confirmed the occurrence of *G.armigerella* in central and western Asia as well as in the Far East of Russia and in China (Fig. 1). The findings from western Tien Shan constitute an important addition to the findings from northern Iran (Forcart 1935). The evidence from northern Tajikistan also fits into this picture. Although these specimens were identified as *G.huttoniana* (Schileyko 1984) they clearly correspond morphologically to *G.armigerella*. Since *G.armigerella* has so far not been found alive in western and central Asia and it is possible that the shells are sub-fossil or fossil, it must be assumed that the species has become extinct there. The dating of the shells, e.g. ^14^C dating, is problematic because the shells are extremely small. Finding the sediment deposits from which the shells were washed out would allow dating of the material with alternative methods. *G.theeli* has also not been found alive in western Tien Shan, but its (sub) fossil occurrence fits with the widespread distribution of relict populations of this species from the Caucasus to the Altay and the Far East.

Some forms of *G.armigerella* which differ slightly in their overall appearance have been described as a subspecies, such as *G.a.hachijoensis* Pilsbry, 1916 from Japan (Hachijojima, Izu, Hirase), *G.a.daitojimana* Kuroda, 1960 also from Japan and, as already mentioned, *G.a.masenderanensis* Forcart, 1935 from northern Iran. These forms cannot be discussed in greater detail here. They probably fall into the synonymy of *G.armigerella*.

Uvalieva (1990) mentioned *Gastrocoptagracilidens* (Sandberger, 1875) from the Pleistocene and Holocene in central Asia (Kazakhstan and the surrounding area) without more precise primary data and *G.huttoniana* from the Pleistocene of that area. *G.gracilidens* is a synonym for *G.nouletiana* (Dupuy, 1850). *G.nouletiana* is very similar to *G.armigerella*, but more compact in appearance and has a larger number of palatal teeth. Moreover, *G.nouletiana* was widespread in Eurasia in the Miocene approximately 15 million years ago (Steklov 1966: 141, fig. 48; Stworzewicz 1999: 164, fig. 59–61). In the case of *G.huttoniana*, it is possible that Uvalieva was referring to the description by Schileyko (1984). In conclusion, it must therefore be assumed that there are possibly further occurrences of *G.armigerella* in central Asia which have merely not yet been interpreted correctly.

Whether *G.coreana* and *G.theeli* are synonyms of each other remains an open question. Likewise, it is currently still unclear whether *G.armigerella* occurs in addition to *G.hirasei* in Korea. These questions can probably be only solved using molecular methods. In addition, it should be checked whether *G.huttoniana*, with its variable shell morphology in the Himalayan region, indeed represents a single taxon.
